# Early Days of Tenascin-R Research: Two Approaches Discovered and Shed Light on Tenascin-R

**DOI:** 10.3389/fimmu.2020.612482

**Published:** 2021-01-08

**Authors:** Fritz G. Rathjen, Russell Hodge

**Affiliations:** Department of Neuroscience, Max-Delbrück-Center for Molecular Medicine, Berlin, Germany

**Keywords:** tenascin-R, tenascin-C, human diseases, perineuronal nets, scaffolding

## The Path to Tenascin-R

It has taken nearly 30 years. But finally, studies on cohorts of patients seem to be shedding some light on a protein that has been surfaced from time to time in the neurobiological literature. Tenascin-R, as it is now known, has proven a slippery quarry. That almost works as a pun, given the fact that in certain contexts, the molecule interferes with cell adhesion.

The protein was first identified in chicken and rodents in the late 1980s among a large number of molecules associated with axons. Significant efforts were being made to untangle the mysteries of axon growth, fasciculation and pathfinding (1). Monoclonal antibody approaches turned up a number of immunoglobulin (Ig)-like cell adhesion proteins (2) which, introduced into cell cultures, influenced the development of neurites (3). One of these was an IgCAM that interacted with the plasma membrane *via* covalently linked glycosylphosphatidylinositol; it was variously termed F11 protein, F3 or contactin—nowadays contactin1 (4–9). Immunoaffinity isolates of contactin1 yielded a complex of at least two polypeptides. The major component was contactin1, at 130 kDa, and along with it a minor partner at about 170 kDa. Further biochemical and immunological experiments showed that the minor component was unrelated to the 130 kDa contactin1, suggesting they had copurified (5, 10).

Antibodies to the 170 kDa protein revealed that it was expressed in the developing nervous system, in a pattern partly overlapping with that of contactin1 but spatially much more restricted. In the spinal cord, for example, it was found on the ventral side around motor neurons during embryonic development. This position suggested a name: restrictin (11). Independently, the same protein was discovered in the Schachner laboratory through different means: using the L2 monoclonal antibody directed to the L2/HNK-1 carbohydrate moiety (12), which captured several glycosylated proteins from neural tissues. These included, IgCAMs, tenascin-C (initially named J1-200/220) and tenascin–R (christened J1-160/180 or janusin by Schachner and her colleagues) (13).

## Structural Features and the Tenascin Family

**Figure 1:**
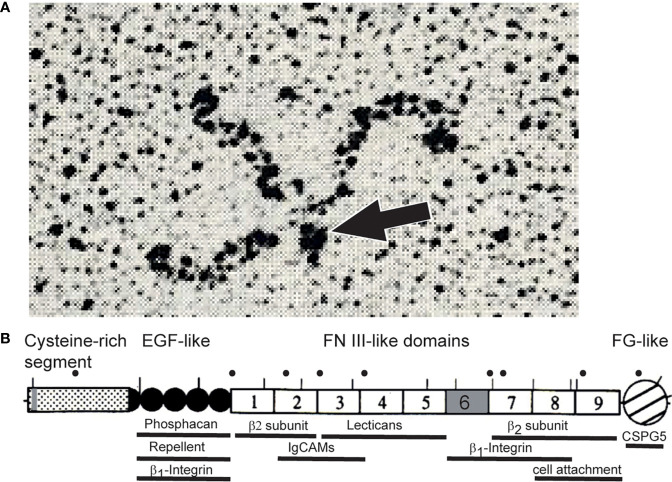
**(A)** Rotary shadowing electron micrograph of tenascin-R purified from brains revealing a trimeric structure. Dimeric and monomeric but no hexameric forms were seen in these electron micrographs (13, 19). The TN-R polypeptide contains a single cysteine amino-terminal to the trimer-forming segment. This cysteine might connect two trimers into hexamers, the hexabrachion structure. It is therefore likely that TN-R might also form hexabrachions in tissues as found for tenascin-C (24). However, alternative splicing or proteolytical cleavage in the N-terminal segment might affect multimer formation of tenascin-R (19, 25). The arrow points to the N-terminal knob formed by a triple-stranded coiled coil. **(B)** Scheme of tenascin-R polypeptide. Lines above the scheme indicate putative N-glycosylation sites of the chicken protein. Human disease mutations are marked by dots above the scheme and regions that bind to cell surface receptors, extracellular matrix proteins or indicate cellular activities are marked by bars below the scheme (please see text). Alternative splice sites of the pre-mRNA encoding TN-R are colored in grey. “β2-subunit” refers to the β2-subunit of sodium channels.

The experiments in chick and rodents had turned up homologs, as became clear through molecular cloning and sequencing of the chick and rat cDNAs. This showed that the components of restrictin and J1-160/180 represented the products of a common gene, and proteins were based on a set of structural motifs found in tenascin-C (14–18). These include an N-terminal cysteine-rich segment with three heptad repeats, followed by 4.5 EGF-like domains, nine fibronectin type III domains, and a C-terminal knob. The latter consists of a globular domain similar to the carboxyl terminal portion of the β- and γ-chains of fibrinogen (19, 20). The N-terminal cysteine-rich region serves as an oligomerization domain. Three heptad repeats of hydrophobic amino acids fold in an α helix and generate a triple-stranded coiled coil to form a trimer which is stabilized by the surrounding cysteines. The related tenascin-C forms hexamers, which may also be the case for tenascin-R (21–23). So far, however, it has only been found as trimers, dimers and monomers in isolates of brain tissues (**Figure 1**) (13, 19). The N-terminal oligomerization domain of the pre-mRNA also contains one alternative splicing site; the 6^th^ fibronectin type III domain is also alternatively spliced. Based on these overall similarities, James Bristow and colleagues suggested renaming restrictin and J1-160/180 to tenascin-R; the proposal was promptly supported in a review article by Harold Erickson (24, 26). Subsequently the nomenclature has been universally adopted. Tenascin-R is the smallest member of the family, and the relationship of its sequence and those to TN-C, TN-X, TN-Y, and TN-W suggests that they arose from a primordial gene that most closely resembled TN-R (26, 27).

## Teasing Out the First Interaction Partners

The complex, modular structure of tenascin-R clearly indicated a potential for diverse molecular interactions, most likely with other proteins on the cell surface. An early goal was to identify receptors that might interact with it and to map regions that could be essential for binding (28). In addition to contactin1, the partner responsible for its discovery, tenascin-R was found in complexes with the IgCAM members neurofascin and contactin2 (previously called axonin-1 or TAG1) (29). An additional cell surface receptor was found in a molecular interaction screen: the transmembrane protein CSPG5 (previously termed CALEB, or neuroglycan C). CSPG5 contains an EGF domain, an acidic stretch and chondroitin-sulfate chains, and it binds to the fibrinogen-like globular domain of tenascin-R (30–34).

More support for a physiological interaction between tenascin-R and contactin1 has come from molecular mapping studies. Immunoglobulin domains 2–4 of the latter molecule are sufficient for the interaction (35), and binding occurs to the second and third fibronectin type III domain of tenascin-R. This region is also important for interactions with neurofascin and contactin2 (29, 36). Tenascin-R interacts with other ECM proteins including fibronectin, β1-integrins (37) and it binds with high affinity to phosphacan and a class of extracellular chondroitin sulfate proteoglycans collectively called lecticans (aggrecan, versican, brevican, and neurocan) (38–43). Later work demonstrated that tenascin-R also contains chondroitinsulfate chains of its own (25, 44).

Most of the interaction partners of tenascin-R have been defined primarily *in vitro* and through cell adhesion assays. Light microscopy work supports the colocalization of tenascin-R with these proteins in some contexts, but for the most part, there is a lack of *in vivo* evidence of direct binding.

## The Search for Biological Functions of Tenascin-R

Cell culture experiments in the early days of tenascin-R research hinted at a number of putative functions through adhesion or neurite outgrowth assays. While many extracellular matrix glycoproteins are known to promote the attachment and spreading of cells, tenascin-R promotes only weak cell adhesion and does not affect cell spreading. For example, the 8^th^ to 9^th^ FNIII domains of tenascin-R serve as a weak cell attachment site for neural cells, which can be specifically blocked by mAb 23-14 (19). In some culture systems, tenascin-R even repels axons or inhibits their regeneration (45–47). A number of *in vitro* studies have also shown that tenascin-R modulates homophilic and heterophilic interactions between IgCAMs and extracellular matrix glycoproteins on neural cells (6, 29, 36, 39, 46, 48–54).

Obviously, the results of such *in vitro* studies have to be taken with a grain of salt, given that they may not accurately reflect the situation in the intact organism or provide true insights into the functions of tenascin-R *in vivo*. Here, further insights can come through studies of expression patterns. TN-R is apparently restricted to the central nervous system, but is absent from the peripheral—with the exception of transient expression on Schwann cells (11, 20, 55–58). Around 2000 came a breakthrough with observations that tenascin-R is localized at perineuronal nets (59, 60). These structures were long known, having been described by several authors at the turn of the 20^th^ century. They surround groups of neurons and synapses on cell bodies primarily in the mature brain, comprising a specialized form of the extracellular matrix; constituents include hyaluronan, lecticans, and several other kinds of CSPGs (61, 62). Perineuronal nets attracted particular interest as brain structures that appear to be implicated in terminating the critical period for neuronal plasticity (63). Here a crucial function for tenascin-R began to emerge; it appears to be essential for the normal development of perineuronal nets. Tenascin-R-deficient mice exhibit an abnormal aggregation of perineuronal CSPGs (59, 64, 65). In electron microscopy images, purified tenascin-R appears to crosslinks aggrecan complexes, which suggests that within the nets, tenascin-R might provide a molecular scaffolding for lecticans (40).

Knockouts of tenascin-R produced a number of phenotypes that might be traceable to this scaffolding function. At the cellular and functional levels, deficiencies lead to mild abnormalities in synaptic transmission and architecture (66, 67). These might arise through disruptions of structures involving lecticans or IgCAMs and CSPG5 (32, 33, 68, 69). Ultimately, the effects on the mature brain involve both structural and functional abnormalities. Consequently, tenascin-R deficient mice exhibit behavioral deficits such as severe impairments in locomotion and hippocampal-associated learning impairments (70).

Another interesting feature of tenascin-R emerged: *in situ* hybridization experiments revealed a dominant colocalization with oligodendrocytes during the period of active myelination (20, 71, 72). In tenascin-R knockout mice, the nodes of Ranvier appear normal, but an analysis of compound action potential recordings from optic nerves revealed a decrease in the conduction velocity. A potential reason for this might be the lack of expression of tenascin-R on oligodendrocytes, which appears to be essential for their differentiation (48, 72); another could be that under normal conditions, the protein might associate with sodium channels to modulate their function (65, 73, 74).

Very recently, a few studies on mouse knockouts combined with cell culture experiments have also shown that tenascin-R modulates the differentiation of neural stem cells during developmental and adult stages. In the olfactory bulb—a structure with a continuous flow of newborn neurons from the subventricular zone of the lateral ventricles—tenascin-R acts as a molecular cue that initiates a radial migration of neuroblasts toward the outer cell layers of the olfactory bulb. Consequently, an absence of tenascin-R affects the recruitment of neuroblasts in the olfactory bulb (37, 75, 76). In the dentate gyrus of the hippocampus, tenascin-R is required for the fate determination of neural stem or progenitor cells. Its absence leads to an increase in the number of GABAergic neurons was increased (77, 78). In summary, these findings point to a role of tenascin-R on neurogenesis.

## Discussion

### Insights From Human Disease Mutations

Recently, an extensive exome sequencing study by a consortium identified 13 patients from eight unrelated families with biallelic variants in the human tenascin-R gene. Combined with two case studies already in the literature, this represents an important chance to investigate functions of the human form of the gene. So far, all of the patients affected have shared some common traits, particularly delays in motor development. The severity varies, ranging from spastic para- or tetraparesis, axial muscular hypotonia, to dyspraxia and transient opisthotonus (79–81). For example, compared to healthy counterparts, patients with the mutation take longer to develop unsupported sitting or standing. In line with these observations are data on mouse knockouts of tenascin-R or its cellular receptor CSPG5, which also exhibit motor deficits (33, 70). These problems in motor development in human patients can most likely be traced to the brain, given current evidence suggesting that tenascin-R expression is primarily restricted to the central nervous system (11, 19, 20, 55–58). And roughly half of the 13 patients revealed mild or moderately impaired cognitive development, including delayed language progression. Once again, there were differences in the degree to which patients were affected.

Overall, tenascin-R associated mutations led to health issues generally considered to be nonprogressive—which is in line with a pattern of expression in which tenascin-R predominantly appears during early brain development (79). MRI of patients’ brains showed delayed myelination consistent with observations on tenascin-R mouse knockouts (48, 72) and, in a few cases, abnormalities in the structure of the corpus callosum. Both observations might be explained if humans follow the pattern observed in the mouse, where tenascin-R is expressed in developing oligodendrocytes (20, 71). The precise effects of the human missense mutations have yet to be determined: whether they affect the overall expression of tenascin-R or interactions with some of the interaction partners mentioned above. Some of these issues may be resolved with further binding assays and the generation of mouse models that replicate mutations specific to the in human patients. It would be particularly interesting to introduce mutations that interfere with molecular interactions of tenascin-R during the formation of perineuronal nets. If this impaired synaptic plasticity or disrupted the normal maturation and maintenance of neuronal circuits during critical periods of brain development, we would stand to learn much about the function of these complex intercellular structures.

As has been the case with many other diseases, the study of mutations in the human tenascin-R gene will likely prove to be a game changer, stimulating research into tenascin-R and clarifying aspects of its functions that go beyond the molecule itself. Insights into its functions could redirect studies performed on the current mouse models. For example, tenascin-R might give scientists a handle on particular brain regions and their complex interactions at highly specific moments in development. The fact that human patients exhibit delays in motor development mean that this approach might serve as a wedge into this highly complex system within the central nervous system. It is interesting that in MRI examinations of the patients, the cerebellum appears normal (79). What would be the result of specifically inactivating tenascin-R in the mouse cerebellum? This might reveal whether the cerebellum plays a role in the axial hypotonia or spasticity observed in patients with mutations in the tenascin-R gene—or whether these deficits are related to changes in perineuronal inhibition at the level of the spinal cord or basal ganglia. At the very least, this might offer a new perspective on how cells and structures in the developing nervous system intertwine at various levels to produce, on the one hand, a marvelously functioning organism—or on the other, a patient burdened by the symptoms of motor diseases.

## Author Contributions

All authors contributed to the article and approved the submitted version.

## Funding

The work of the author was supported by the Max-Delbrück-Center and grant SFB665 by the DFG. 

## Conflict of Interest

The authors declare that the research was conducted in the absence of any commercial or financial relationships that could be construed as a potential conflict of interest.
